# Construction of a high-density genetic map and fine QTL mapping for growth and nutritional traits of *Crassostrea gigas*

**DOI:** 10.1186/s12864-018-4996-z

**Published:** 2018-08-22

**Authors:** Chunyan Li, Jinpeng Wang, Kai Song, Jie Meng, Fei Xu, Li Li, Guofan Zhang

**Affiliations:** 1 Key Laboratory of Experimental Marine Biology, Institute of Oceanology, Chinese Academy of Sciences, Qingdao, China; 2 University of Chinese Academy of Sciences, Beijing, China; 3 Laboratory for Marine Biology and Biotechnology, Qingdao National Laboratory for Marine Science and Technology, Qingdao, China; 4 Laboratory for Marine Fisheries and Aquaculture, Qingdao National Laboratory for Marine Science and Technology, Qingdao, China; 5 Center for Ocean Mega-Science, Chinese Academy of Sciences, Qingdao, China; 6 National & Local Joint Engineering Laboratory of Ecological Mariculture, Chinese Academy of Sciences, Institute of Oceanology, Qingdao, China

**Keywords:** *Crassostrea gigas*, Genetic map, Collinearity, Quantitative trait loci (QTL) mapping, Growth, Nutrition

## Abstract

**Background:**

Both growth and nutritional traits are important economic traits of *Crassostrea gigas* (*C. gigas*) in industry. But few work has been done to study the genetic architecture of nutritional traits of the oyster. In this study, we constructed a high-density genetic map of *C. gigas* to help assemble the genome sequence onto chromosomes, meanwhile explore the genetic basis for nutritional traits via quantitative trait loci (QTL) mapping.

**Results:**

The constructed genetic map contained 5024 evenly distributed markers, with an average marker interval of 0.68 cM, thus representing the densest genetic map produced for the oyster. According to the high collinearity between the consensus map and the oyster genome, 1574 scaffold (about 70%) of the genome sequence of *C. gigas* were successfully anchored to 10 linkage groups (LGs) of the consensus map. Using this high-qualified genetic map, we then conducted QTL analysis for growth and nutritional traits, the latter of which includes glycogen, amino acid (AA), and fatty acid (FA). Overall, 41 QTLs were detected for 17 traits. In addition, six candidate genes identified in the QTL interval showed significant correlation with the traits on transcriptional levels. These genes include growth-related genes *AMY* and *BMP1*, AA metabolism related genes *PLSCR* and *GR*, and FA metabolism regulation genes *DYRK* and *ADAMTS*.

**Conclusion:**

Using the constructed high-qualified linkage map, this study not only assembled nearly 70% of the oyster genome sequence onto chromosomes, but also identified valuable markers and candidate genes for growth and nutritional traits, especially for AA and FA that undergone few studies before. These findings will facilitate genome assembly and molecular breeding of important economic traits in *C. gigas*.

**Electronic supplementary material:**

The online version of this article (10.1186/s12864-018-4996-z) contains supplementary material, which is available to authorized users.

## Background

The Pacific oyster, *Crassostrea gigas* (*C. gigas)* originated in Asia, and has become an important cultured mollusc in many countries as a result of its rapid growth rate and high yield [1, 2]. In 2016, the global aquaculture production of *C. gigas* was estimated to be 625,925 metric tons with a value of approximately 1.34 billion dollars (FAO, 2016). As an edible aquatic product, quality and flavour plays a key role in the commercial value of *C. gigas*. Growth and nutritional traits are not only associated with the oyster production but also important indicators of oyster quality. Oysters are rich in glycogen, essential amino acids (EAAs), unsaturated fatty acids (UFAs), and vitamins [3]. High levels of nutrients give the oyster high physiological and medicinal value. For example, glycogen from the oyster meat can be adsorbed directly by the human body, thus lighten the burden of the pancreas [4]. As a sulphur-containing neurotransmitter, taurine (Tau) plays a key role in the development of the nervous and cardiovascular systems [5, 6], and is involved in many physiological and metabolic processes [7, 8]. The protein content of the oyster dry meat weight was reported as high as 57%, and the quality and composition of its EAAs were found to be superior to the milk [3, 4]. In addition, the UFAs of the oyster meat, such as C20:5ω3 (EPA) and C22:6ω3 (DHA), were reported to prevent cardiovascular and cerebrovascular diseases [9, 10]. Because of its commercial and industrial importance, breeding work has been performed to improve the quality of *C. gigas.* Compared with the growth traits, which have got great attention in previous phenotype-dependent selective breeding programs [11–14], few nutritional traits of *C. gigas,* other than glycogen [15], have been subjected to molecular breeding. Though nutritional traits of the oyster were reported to vary with the environment, like the season and sea area [16, 17], the oyster family cultured in the same environment exhibited great phenotype variation of the nutritional traits (Additional file 2, [18]), which indicated obvious genetic effect for nutritional traits.

Linkage analysis is an important tool for quantitative trait loci (QTL) mapping, marker-assisted selection (MAS) programs, and genome assembly. For the oyster, a total of 20 articles have reported studies concerning genetic map construction or QTL mapping [19–38]. To date, QTLs for growth [31, 36], glycogen [34, 37], disease resistance [24, 29], and shell color [34, 38] traits have been detected in previous studies. However, no QTLs for AA and FA traits have been detected before and reported maps did little for the assembly of the oyster genome because of the absence of the genome or enough markers. In this study, we selected the double-digest genotyping by sequencing (ddGBS) method to genotype our full-sib mapping family. With thousands of polymorphic loci detected, we constructed a high-density genetic map. We not only evaluated the collinearity between the genetic map and the genome of *C. gigas*, but also conducted QTL mapping for many economic traits, including AAs and FAs that had never undergone QTL mapping or association analysis in this species. This work assists to assemble the genome sequence of *C. gigas* onto chromosomes, meanwhile help illuminate the genetic mechanism of important economic traits so as to promote MAS programs of *C. gigas*.

## Methods

### Mapping population, DNA and RNA extraction, and cDNA cloning

In July 2013, the mapping family was constructed by mating one maternal oyster (Changli, China) with another paternal oyster (Qingdao, China). All oysters were pasted with different labels on the shell to distinguish from natural spat before culturing in the sea in September 2013. In January 2015, a total of 169 F1 offsprings were randomly selected from this family as the mapping population. Mantles of the oysters were stored at − 80 °C for DNA and RNA extraction and the other tissues were sampled and stored at − 20 °C for nutrients detection. DNA was extracted with a DNeasy® Blood & Tissue Kit (QIAGEN, Germany) according to the user’s instructions. RNA was extracted using TRIzol reagent (Invitrogen, USA) according to manufacturer’s protocol. cDNA was reverse-transcribed from 1 μg of total RNA in a 20-ul reaction mixture using PrimeScript RT reagent kit with gDNA Eraser (TaKaRa, Japan) following the manufacturer’s instructions. All experiments were conducted with approval from the Experimental Animal Ethics Committee, Institute of Oceanology, Chinese Academy of Sciences, China.

### Phenotype detection and analysis

Growth and nutritional traits were analysed for all 169 full-sib offsprings. Growth traits include shell height (SH), shell length (SL), shell width (SW), body weight (BW) and weight of soft tissue (STW), while nutritional traits include glycogen, 18 AAs, and 22 FAs. All nutritional traits were detected using the dry soft tissue that was dried with the freeze dryer (Boyikang, China). Glycogen content was detected using a liver/muscle glycogen assay kit (Nanjingjiancheng, China) according to the instructions. AAs were detected using an automatic amino-acid analyser LC-3000 (Eppendorf-Bio-Tronik, Germany) according to Chinese national standard GB/T 18246–2000. FAs were detected and analysed using a gas chromatograph (GC, Agilent 7890A) method [39] with slight modifications. To better understand the characteristics of detected traits, histogram distribution, and Pearson’s correlation analyses were performed using R 3.2.3.

### GBS library preparation and sequencing

Before carrying out the experiment, Perl package RestrictionDigest [40] was used to evaluate the number of restriction sites, and the *Aci*I-*Bfa*I restriction enzyme-cut combination was selected. After the DNA concentrations of 171 individuals (two parents and 169 full-sib progenies) were quantified to 20 ng/μL by Qubit 2.0 (Thermo Fisher Scientific, Waltham, MA), the GBS library was constructed with slight modifications to the reported method [36]. In short, the DNA quantities of two parents were three-times that of the progeny. Adapter 1 and 2 containing the enzyme-cut site, barcode, and primer sequence matches *Aci*I and *Bfa*I, respectively. The length of the barcode ranged from 4 to 13 bp. A total of 9 kinds of adapter 1 and 10 kinds of adapter 2, were used (Additional file 1). Primers used for PCR amplification are also listed in Additional file 1. Pair-end 125-bp (125PE) sequencing of the library was performed on the Illumina HiSeq 2500 system (Illumina, San Diego, CA) according to the manufacturer’s recommendations.

### Quality control and genotyping of sequencing data

Raw reads were first filtered to clean reads with the following criteria: reads with adapter sequences, unreliable reads (> 10% ‘N’ in the sequence), and low-quality reads (> 50% positions with quality < 5) were removed. After all reads were trimmed to 112 bp using a Perl script, Stacks software was used to distinguish and genotype the mapping population [41]. First, the command “process_radtags -c -q -r --inline_inline --renz_1 aciI --renz_2 bfaI -i gzfastq” was run to demultiplex lane data to one fastq file per individual. In this step, reads of low quality, reads with wrong enzyme-cut sites, or with incorrect barcodes were discarded. Second, pair-end reads of each individual were aligned to an updated Pacific oyster genome (V9.2, in preparation) using the BWA aln parameter to produce a “sam” file [42]. Based on the sam file, reads aligned to multiple genome positions were deleted by the “grep -v ‘XA:Z’” command, and badly aligned reads (align score < 20) were removed with a Perl script. After that, pstacks, cstacks, sstacks, and genotype procedure of Stacks were run in turn to build and genotype the haplotype marker across the full-sib family. In summary, only markers with a sequencing depth over 5× were reserved. We further filtered for the missing value rate (> 90%, that means genotyped in more than 152 individuals) for each of the marker. The output type was set as the CP population towards JoinMap4.1 software.

### Linkage map construction and genome collinearity analysis

Prior to map construction, a segregation distortion test was performed. There are four categories of markers according to the segregation patterns: lm x ll (markers from the male parent with a 1:1 segregation ratio in the family), nn x np (markers from the female parent with a 1:1 segregation ratio in the family), and ef x eg and ab x cd (markers from both parents with 1:1:1:1 segregation ratio in the family). Markers showing significant segregation distortion with *P* < 0.01 in Chi-square goodness-of-fit tests were excluded. Sex-specific and averaged linkage maps were constructed using JoinMap4.1 [43]. Markers were assigned to different LGs by a LOD threshold of 5.0. Marker distance was calculated using haldane function within the maximum likelihood (ML) algorithm. Sex averaged map was drawn using Mapchart 2.3 [44].

To evaluate the collinearity between the genetic map and genome assembly, the sequences of the markers, which were extracted from the tsv file produced by cstacks, were mapped onto the genome of *C. gigas*. We quantified the number of scaffold that contains at least two markers, and then analysed the distribution of the markers on the same scaffold.

### QTL mapping of growth and nutritional traits

MapQTL6.0 software was employed to detect QTLs for all detected traits using interval mapping (IM) and restricted multiple QTL model (rMQM) [43]. The LOD threshold was set by 3000-times-permutation tests at *P* < 0.05 for each trait. Mapping step size was set to 0.1 cM, and only QTLs with a LOD score exceeding the threshold were regarded as significant. To further confirm the QTL mapping results, we conducted association analysis between traits and genotypes using Tassel 5.0 with the MLM (PCA + K) method [45]. Considering the results from both QTL mapping and association analysis, we identified markers that were significantly correlated with the traits, and identified candidate genes around these markers utilising the reference genome and annotation file of *C. gigas*.

### Quantitative gene expression analysis of candidate genes

For each gene identified in the QTL, we firstly genotyped the full-family by the nearest marker of the gene. Then we compared the phenotypic values of the individuals that with different genotypes to ensure that the marker was indeed associated with the trait. Samples with the genotype that showed higher and lower phenotypic values were then named ‘high group’ and ‘low group’, respectively. Based on the genotyping results, we selected eight samples with the highest phenotypic values from ‘high group’ and eight samples with the lowest phenotypic values from ‘low group’, respectively. Further, we compared the gene mRNA expression levels between the two groups. The real-time reverse transcript polymerase chain reaction (qRT-PCR) experiment was performed using SYBR® Premix Ex Taq™ II (Tli RNaseH Plus) (TaKaRa, Japan) on ABI 7500 Fast Real time Thermal Cycler (Applied Biosystems, USA) according to the instructions. The expression levels of the gene were calculated with the 2^-△△CT^ method. Besides, elongation factor (EF) primers were used as the internal control primers [18], and a random sample from the ‘high group’ was taken as the reference sample. All the primers used in this study were listed in Additional file 1.

## Results

### Phenotype analysis

Additional file 2 shows phenotypic values and variations for growth and glycogen, AA, and FA traits of 169 full-sib progenies. The distribution of different traits displayed various patterns with abundant variations, e.g. glycogen content ranged from 26.5 to 217.24 mg/g, representing a difference of more than 8 fold; the total AA content (All_AA) ranged from 27.38 to 56.78%; and the total FA content (All_FA) ranged from 16.9 to 58.9 mg/g (Additional file 3). Among the 18 detected AAs, glutamic acid (Glu) and Tau exhibited the highest levels, accounting for 15.21 and 11.94% of the total AA content, respectively (Additional file 4 A). Twenty-two fatty acids were detected, C16:0 exhibited the highest level, accounting for 47.82% of the total FA content (Additional file 4 B). We also conducted phenotypic correlation analysis for all traits. The results indicated that growth and glycogen were positively correlated with each other (Additional file 5 A), and the same result was also shown by AAs (Additional file 5 B) and FAs (Additional file 5 C) correlation pattern. Furthermore, by selecting representative traits from each major category, we found that glycogen, growth, and FA traits were positively correlated with each other, and they were all negatively correlated with AA traits (Additional file 5 D).

### GBS library sequencing and marker genotyping

After reads were demultiplexed and cleaned using Perl scripts and process_radtags program, a total of 860.7 million (M) clean reads were generated. There were, on average, 7.54 and 4.3 M clean reads for parents and progeny, respectively, which corresponded to an actual sequencing depth of 27× on average. Using data from the parents, a catalogue of 296,187 markers was built. Based on this catalogue, 55,792 polymorphic markers with more than 5× sequencing depth were identified across all the samples. We calculated the missing rate for all the polymorphic markers, and found that most of the markers (59.63%) were missing in more than half of the population (Additional file 6). To insure the quality of the markers, we only used 7075 markers with the missing rate of less than 10%, and further filtered the markers with the segregation distortion test (*P* < 0.01). In total, 5094 markers were retained for the genetic map construction.

### High-resolution genetic map construction and analysis of recombination rate

**Fig. 1:**
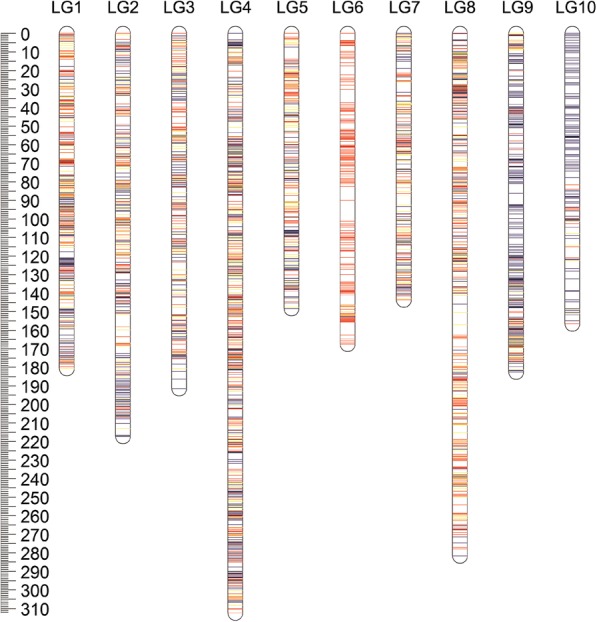
High-density consensus linkage map for *Crassostrea* ***gigas*.** For each linkage group, red, blue, and yellow lines, represent paternal heterozygous markers, maternal heterozygous markers, and markers heterozygous in both parents, respectively

Overall, 5024 markers were successfully assigned to 10 LGs by LOD 5.0 using JoinMap4.1 (Table 1). Detailed information of the markers can be seen in Additional file 7. For sex-specific maps, the paternal map consisted of 2993 markers with a total length of 1765.49 cM, whereas the maternal map consisted of 2963 markers with a total genetic map length of 1931.26 cM (Additional file 8). Using 932 shared markers (ef x eg and ab x cd, which are heterozygous in both parents), a consensus genetic map was constructed. Different types of markers were evenly distributed across the map, with the exception of LG6 and LG10. LG6 contained more paternal markers, while LG10 consisted mainly of maternal markers (Fig. 1). The genetic length of each LG ranged from 148.32 (LG5) to 312.43 (LG4) cM, with the total map length of 1982.07 cM. All 5024 markers of the consensus map occupied 2931 different positions, and the unique marker interval (interval only for markers occupied different positions on the map) ranged from 0.53 (LG1) to 1.22 cM (LG10), with an average interval of 0.68 cM (Table 1).

**Table 1:** Summary of the consensus map of *Crassostrea gigas*

LG	No. of Markers	Length (cM)	Marker interval	^a^No. of unique loci	^b^Average interval of unique loci	^c^No. of shared markers	No. of SNPs
1	530	180.64	0.34	343	0.53	115	1192
2	490	217.33	0.44	300	0.73	109	1159
3	409	191.53	0.47	246	0.78	78	903
4	951	312.43	0.33	583	0.54	212	1914
5	394	148.32	0.38	221	0.67	83	893
6	410	167.54	0.41	176	0.96	10	832
7	361	143.66	0.4	212	0.68	98	847
8	696	281.67	0.41	431	0.66	152	1490
9	490	182.48	0.37	290	0.63	63	1012
10	293	156.48	0.54	129	1.22	12	536
All	5024	1982.07	0.39	2931	0.68	932	10,778
Average	502.40	198.21	0.39	293.10	0.68	93.20	1077.80

^a^Number of loci that occupied different positions on the map

^b^Average interval of loci which occupied different positions on the map

^c^Number of markers that were heterozygous in both parents

**Fig. 2:**
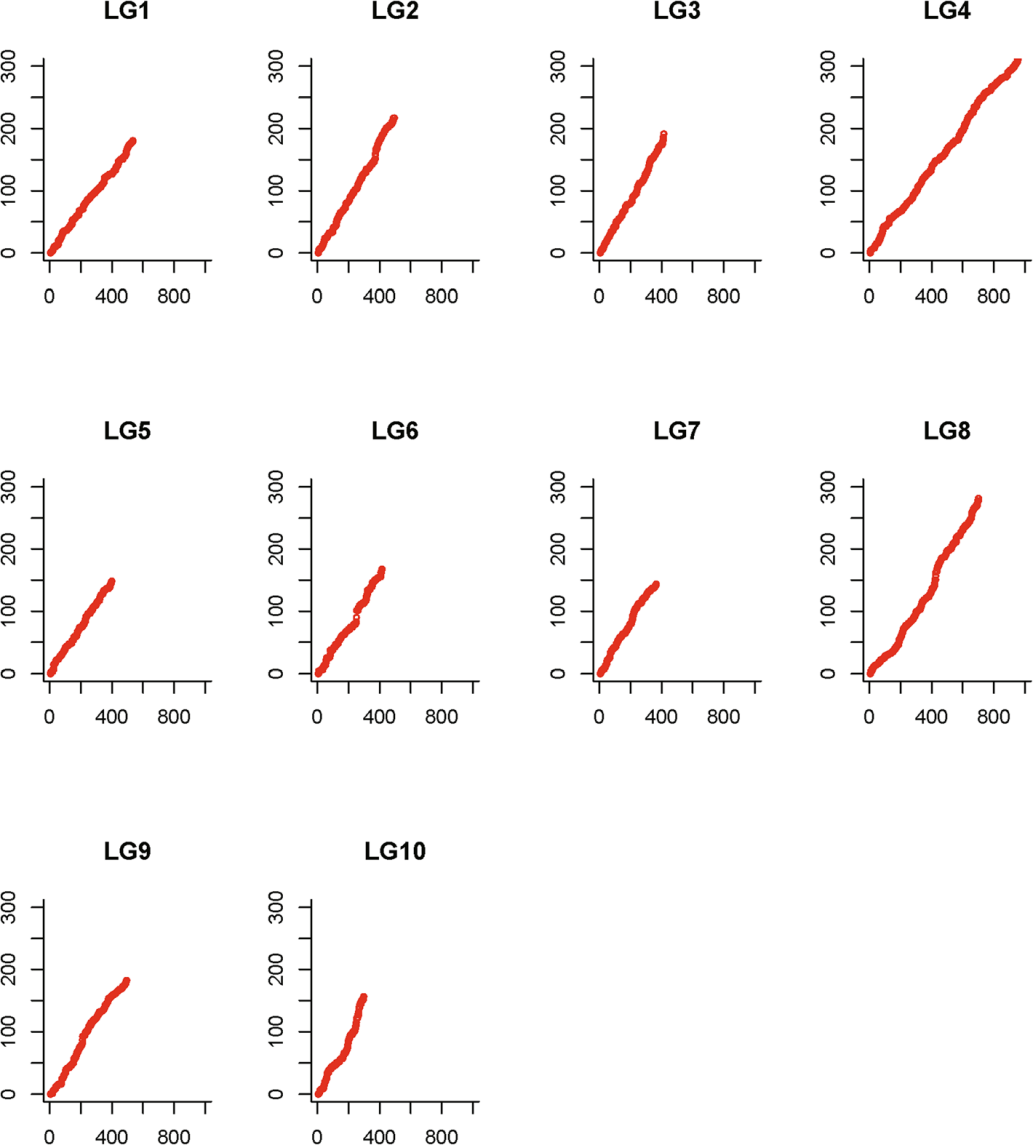
Marker distribution pattern on each linkage group. The X-axis represents marker orders on each linkage group, while the Y-axis represents marker position (cM) on each linkage group

**Fig. 3:**
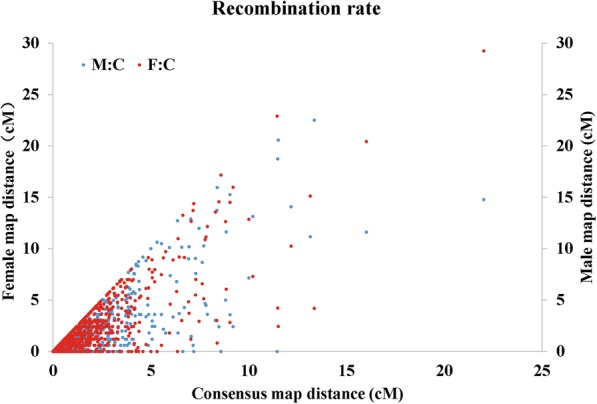
Recombination rates of shared markers between sex-specific maps and the consensus map of *C. gigas*. The X-axis, left-Y axis, and right-Y axis represents the shared marker interval on the consensus map, female map, and male map, respectively. The red and blue plots represent shared marker interval ratios between the female map and consensus map (F:C ratio), and between the male map and consensus map (M:C ratio), respectively

Marker distribution pattern on each LG showed a similar slope among different LGs, which indicated similar recombination rates of different LGs and evenly spaced markers on the consensus map (Fig. 2). Similar recombination rates were also confirmed by comparisons between sex-specific maps. First, the maternal map showed slightly higher values than the paternal map concerning both average map length and average marker interval (the average female-to-male ratio was 1.14 and 1.18, respectively), but these differences were not significant (t-test, *P* > 0.05) (Additional file 8). Second, as marker interval was closely linked to recombination rate, we used the interval of 932 shared markers to evaluate recombination rates between the sex-specific maps. Recombination rates of shared markers between sex-specific maps and the consensus map of *C. gigas* revealed similar recombination rates and high consistency between sex-specific maps (Fig. 3). The average female-to-male ratio for recombination rates of shared markers among different LGs was 1.07, ranging from 0.43 (LG6) to 1.41 (LG2) (Additional file 8). Recombination rates of the female-specific map were slightly higher than those of the male, although no significance was found (*t*-test, *P* > 0.05). High consistency between sex-specific maps laid the basis for generating a high-qualified consensus map.

### Genome collinearity analysis

**Fig. 4:**
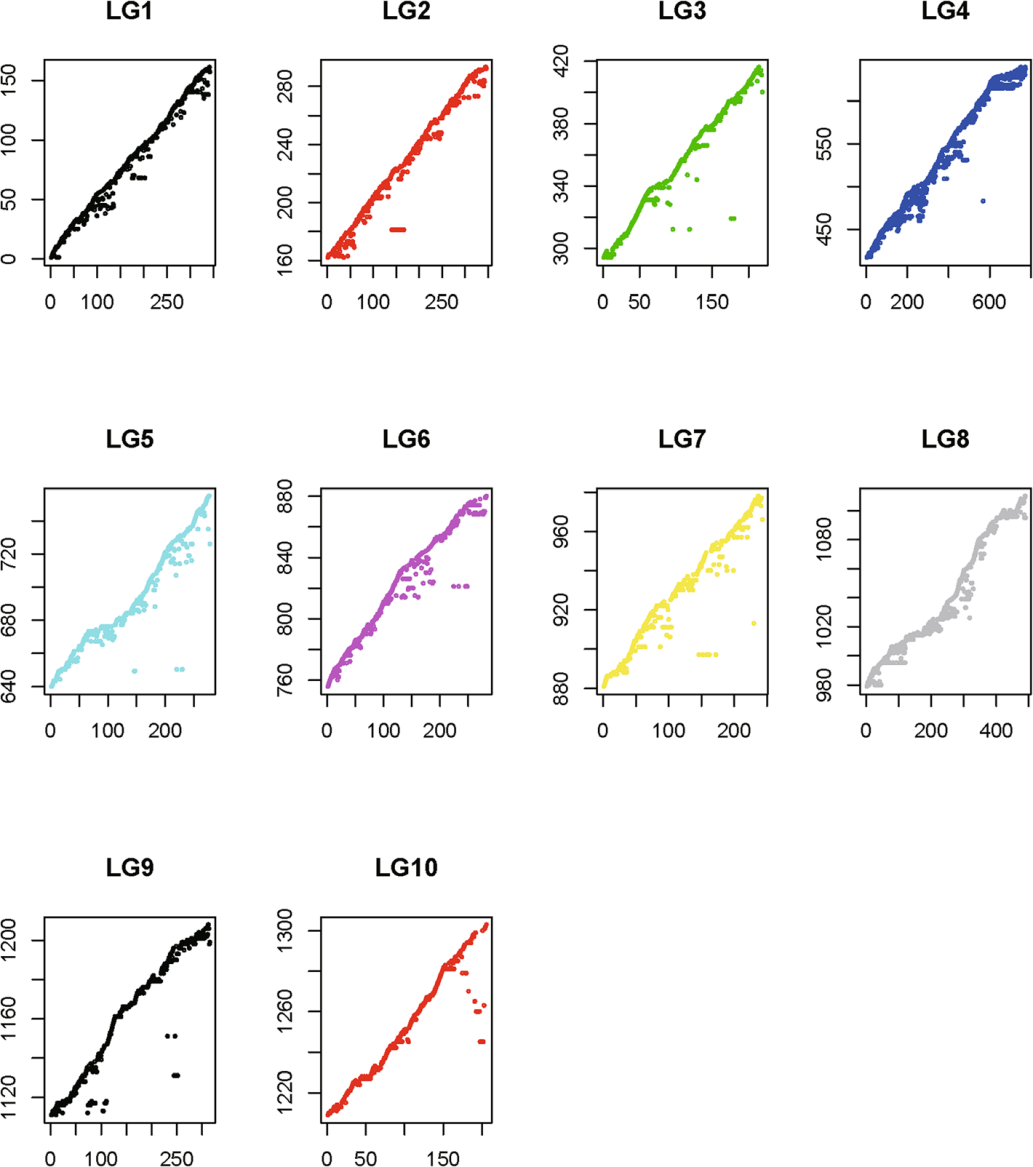
Collinearity analysis between the consensus map and the genome scaffold of *C. gigas*. For each linkage group, the X-axis represents the marker position (cM) on the linkage group, and the Y-axis represents the genome position of the marker (the scaffold mapped by the marker)

Based on the high-qualified consensus map, we used the genome position of the markers to evaluate the collinearity between the map and genome of *C. gigas*. Results showed that all 5024 markers were aligned to 1574 scaffold, covering 68.81% of the oyster genome. Overall, 3809 markers with a unique genome position and an alignment length of over 100 bp were used for further analysis. These 3809 markers were distributed on 1303 scaffold of the genome, and 761 of those scaffold had more than one marker. Overall, 86.47% of the scaffold with at least two markers (658 of 761) were located on the same LG of the consensus map (Table 2). Collinearity analysis of the scaffold position on the genome towards the marker position on the consensus map showed that most plots were on the diagonal or adjacent position, which indicated that markers of the same scaffold were distributed on the same or adjacent position of the consensus map (Fig. 4).

**Table 2:** Scaffold distribution on the consensus map of *Crassostrea gigas*

^a^No. of LGs	^b^No. of scaffold	^c^Ratio
1	658	86.47%
2	92	12.09%
3	9	1.18%
4	1	0.13%
5	1	0.13%
6	0	0.00%
All	761	100%

^a^Number of linkage groups that markers on the same scaffold span

^b^Number of scaffold whose markers show different LG distribution

^c^Percentage of b

### QTL mapping of growth and nutritional traits and candidate genes expression levels analyses

**Fig. 5:**
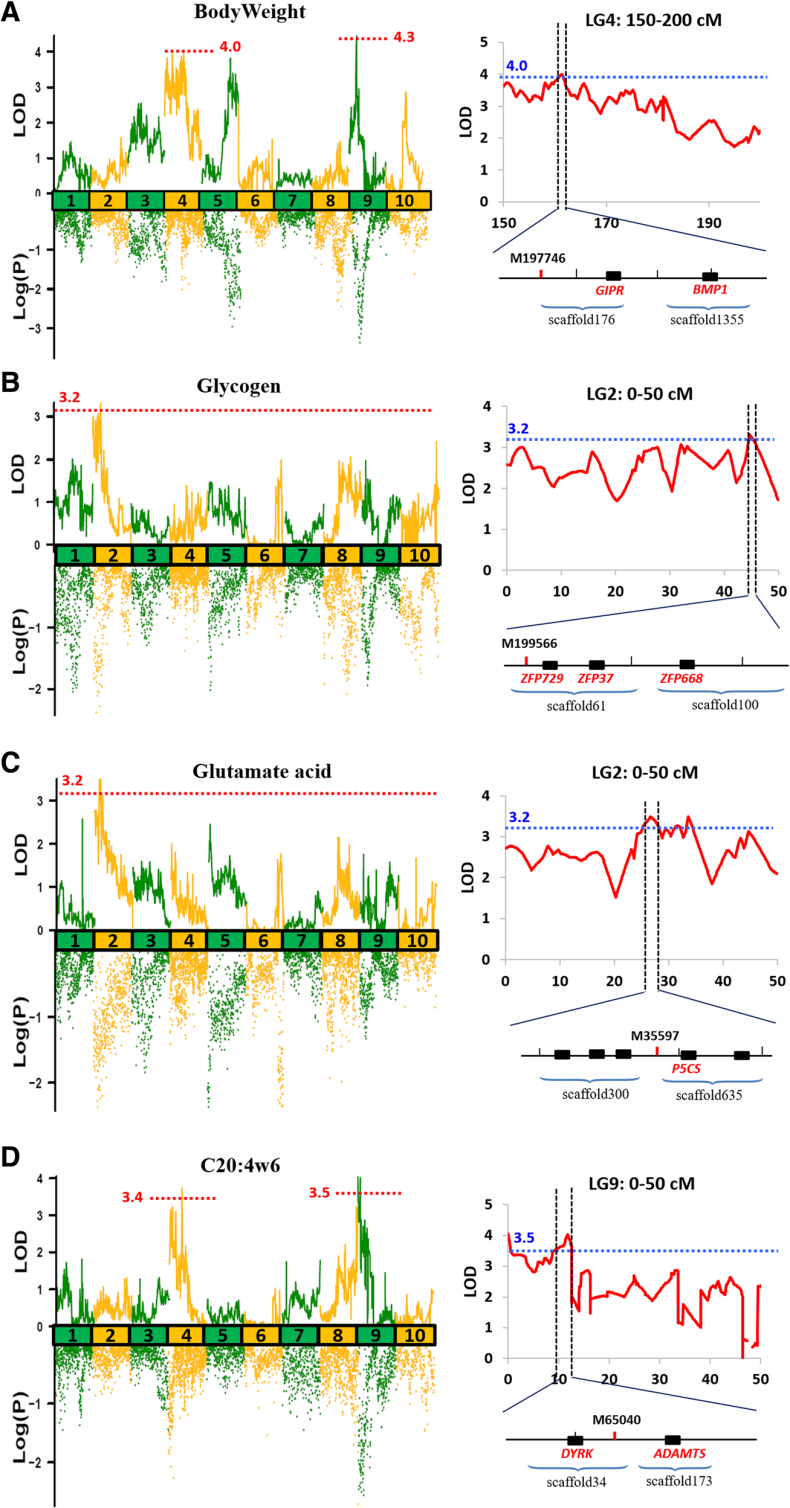
QTL mapping and association analysis for growth and nutritional traits of *C. gigas.* **a** Body weight, **b** Glycogen, **c** Glutamate acid, and **d** C20:4ω6. For the right pattern, the nearest marker of peak LOD loci for each QTL is marked by a short red line; candidate genes identified in the QTL are marked with small black boxes

We detected a total of 41 QTLs for 17 of the 46 traits (Table 3). For growth traits, 15 QTLs were detected for SL, BW, and STW, with the explained phenotype variation (PVE) ranging from 8.9 to 15.2% (Table 3, Fig. 5a, Additional file 9). The correlation coefficient between BW and STW was 0.8 (*P* < 0.01, Additional file 5 A), and all of the QTLs were on LG4 and LG9 (Table 3). Two protein-coding genes were identified in qBW_2 (Table 4, Fig. 5a). Bone morphogenetic protein 1 (*BMP1*) is a metalloproteinase that can regulate the formation of the extracellular matrix and dorsal-ventral patterning in early embryos [46, 47]; While glucose-dependent insulinotropic receptor (*GIPR*), which is responsible for insulin secretion, has been reported to be associated with obesity and weight gain [48–50]. Two genes associated with growth (Tables 3-4) were also identified in on QTL of STW on LG4 (qSTW_4). Alpha-amylase (*AMY*) has been widely reported to affect growth, e.g. AMY inhibits the growth of *Porphyromonas gingivalis* [51], and polymorphisms in *AMY* was correlated with growth traits in *Litopenaeus vannamei* [52]; While fibroblast growth factor receptor (*FGFR*), was found to be significantly correlated with four growth traits in common carp [53].

**Table 3:** QTLs detected for growth and nutritional traits

Traits	QTL name	LG	CI (cM)	Peak LOD	PVE	No. of Markers	Nearest marker
SL	qSL_1	5	107.033~ 107.333	5.62	14.2	2	M80051
	qSL_2	5	107.549~ 121.838	6.04	15.2	40	M215101
	qSL_3	5	125.066~ 126.869	5.13	13	10	M52
	qSL_4	5	141.512~ 144.699	4.89	12.5	6	M217955
BW	qBW_1	4	62.284~ 66.37	4	8.9	17	M32308
	qBW_2	4	157.64~ 164.046	4	8.9	19	M197746
	qBW_3	9	34.627~ 38.127	4.44	9.4	1	M249891
STW	qSTW_1	4	4.116~ 7.9	4.93	11.7	22	M209141
	qSTW_2	4	8.066~ 13.457	5.5	12.9	8	M372
	qSTW_3	4	14.513~ 27.436	5.41	12.7	28	M74592
	qSTW_4	4	42.265~ 43.294	4.57	10.9	15	M101944
	qSTW_5	4	64.117~ 65.155	4.71	11.2	5	M32308
	qSTW_6	4	96.226~ 98.411	5.06	12	3	M44770
	qSTW_7	4	129.586~ 129.586	4.58	10.9	1	M174634
	qSTW_8	9	36.327~ 38.127	4.71	10.8	0	M45417
Glycogen	qGlycogen	2	44.558~ 45.263	3.32	8.6	3	M199566
C17:1	qC17:1	5	60.774~ 61.417	3.41	8.9	1	M25425
C20:1w9	qC20:1w9	6	138.556~ 138.656	3.91	10.2	1	M2829
C20:2	qC20:2	1	150.995	14.13	32.1	0	M234054
C20:3w6	qC20:3w6	7	85.33~ 92.16	3.89	9	4	M87086
C20:4w6	qC20:4w6_1	4	103.477~ 105.036	3.74	8.4	1	M71739
	qC20:4w6_2	9	0~ 0.4	4.04	9.1	1	M221503
	qC20:4w6_3	9	9.176~ 12.534	4.02	9.1	5	M65040
C22:0	qC22:0	1	150.995	24.46	48.9	5	M234054
Glu	qGlu_1	2	25.152~ 28.233	3.49	9.1	6	M35597
	qGlu_2	2	31.11~ 32.179	3.27	8.5	16	M182888
	qGlu_3	2	33.328~ 34.413	3.49	9.1	4	M132222
Thr	qThr_1	2	25.694~ 28.433	3.41	8.9	4	M35597
	qThr_2	2	29.2~ 29.437	3.21	8.4	1	M33164
	qThr_3	2	31.28~ 32.279	3.28	8.6	16	M156218
	qThr_4	2	33.176~ 34.213	3.39	8.8	5	M132222
Val	qVal_1	2	25.894~ 28.033	3.46	9	4	M35597
	qVal_2	2	31.88~ 32.179	3.32	8.7	15	M156218
	qVal_3	2	33.328~ 34.113	3.44	9	4	M132222
Tau	qTau_1	5	9.65~ 14.197	3.38	8.1	2	M114792
	qTau_2	5	15.054~ 15.565	3.3	7.9	5	M83928
	qTau_3	8	134.412~ 135.019	3.35	8.1	1	M219081
	qTau_4	8	196.652~ 197.681	3.76	9	5	M238135
Gly	qGly	6	153.667~ 153.867	3.47	8.3	1	M120451
His	qHis	8	113.985~ 114.885	3.32	8.7	0	M101722
Pro	qPro	6	128.883~ 131.357	5.18	13.2	1	M118734

PVE, explanation of phenotype variance

**Table 4:** Summary of candidate genes detected for significant QTLs of growth and nutritional traits of *C. gigas*

Traits	QTL name	LG	Scaffold ID	Gene name	Gene Anotation
BW	qBW_2	4	scaffold1355	BMP1	Bone morphogenetic protein 1
BW	qBW_2	4	scaffold176	GIPR	Glucose-dependent insulinotropic receptor
STW	qSTW_4	4	scaffold2396	AMY	Alpha-amylase
STW	qSTW_4	4	scaffol539	FGFR	Fibroblast growth factor receptor
Glycogen	qGlycogen	2	scaffold61	ZFP37	Zinc finger protein 37
Glu	qGlu_1	2	scaffold635	P5CS	Delta-1-pyrroline-5-carboxylate synthase
Thr	qThr_1	2	scaffold635	P5CS	Delta-1-pyrroline-5-carboxylate synthase
Val	qVal_1	2	scaffold635	P5CS	Delta-1-pyrroline-5-carboxylate synthase
Tau	qTau_2	5	scaffold202	SDH	Succinate dehydrogenase
Tau	qTau_4	8	scaffold504	GP	glycogen phosphorylase
Tau	qTau_4	8	scaffold504	rBAT	Neutral and basic amino acid transport protein
C17:1	qC17:1	5	scaffold124	PLSCR	phospholipid scramblase
C20:1ω9	qC20:1ω9	6	scaffold1	GHSR	growth hormone secretagogue receptor
C20:4ω6	qC20:4ω6_3	9	scaffold34	DYRK	Dual specificity tyrosine-phosphorylation-regulated kinase
C20:4ω6	qC20:4ω6_3	9	scaffold173	ADAMTS	A disintegrin and metalloproteinase with thrombospondin motifs

For glycogen, only one QTL on LG2 with a PVE of 8.6% was detected (Table 3, Fig. 5b). This QTL interval identified many zinc finger proteins (*ZFPs*), such as *ZFP729*, *ZFP668*, and *ZFP37* (Table 4). ZFP is a transcription factor that functions in many important physiological processes by regulating gene transcription [54]. Of the three types of identified ZFPs, *ZFP37* might be the key gene for its role in the regulation of spermiogenesis [55]. Glycogen metabolism was widely reported to regulate sperm motility and capacitation [56–59]. Therefore, we suggest that ZFP37 might be an upstream regulatory gene in glycogen metabolism, and may regulate spermiogenesis by influencing the glycogen content.

Seventeen QTLs were detected for 7 AAs, including Glu, threonine (Thr), glycine (Gly), Tau, histidine (His), proline (Pro), and valine (Val) (Table 3, Fig. 5c, Additional file 9). The correlation coefficient between Glu, Thr, and Val exceeded 0.94 (*P* < 0.01, Additional file 5 B), and similar QTLs ranging from 25.152 to 34.413 cM were detected for these AAs on LG2 (Table 3, Fig. 5c, Additional file 9). One key gene identified in the QTL was delta-1-pyrroline-5-carboxylate synthase (*P5CS*) (Table 4). P5CS catalyses the reduction of Glu to delta1-pyrroline-5-carboxylate (P5C), a critical step in the de novo biosynthesis of Pro, ornithine, and arginine [60], and is thus a vital enzyme in Glu and other AA metabolism. Two genes related to glycometabolism were identified in qTau_2 and qTau_4, respectively (Table 4). Succinate dehydrogenase (*SDH*) can catalyse the oxidation of succinate to fumarate, with the reduction of ubiquinone to ubiquinol during the citric acid cycle [61]; While glycogen phosphorylase (*GP*) catalyses the rate-limiting step in glycogenolysis in animals by releasing glucose-1-phosphate from the terminal alpha-1,4-glycosidic bond [62]. Tau was reported to have hypoglycaemic effect in previous studies [8, 63, 64]. Therefore, we suggest that Tau may affect glucose levels by regulating enzymes in the metabolic pathway. Furthermore, we identified a neutral and basic amino acid transport protein (*rBAT*) in qTau_4 with a PVE of 9% (Tables 3-4). rBAT functions as cysteine transporter [65–67]. As a sulfur-containing amino acid, cysteine is a substrate of tau synthesis. Therefore, rBAT may influence Tau levels by regulating cysteine transport.

A total of eight QTLs were detected for six FAs (C17:1, C20:1ω9, C20:2, C20:3ω6, C20:4ω6, and C22:0). Eight QTLs detected for FAs were distributed on six LGs, which indicated that different genes might be associated with different kinds of FAs (Table 3, Fig. 5d, Additional file 9). For C20:4ω6, one QTL located on LG4 and two QTLs located on LG9 were detected (Table 3, Fig. 5d). Two genes identified in qC20:4ω6_3 were shown to be associated with body weight or fat mass (Table 4). Dual-specificity tyrosine-phosphorylation-regulated kinase (*DYRK*), can prevent metabolic disorders like obesity by reducing fat mass [68]; while a disintegrin and metalloproteinase with thrombospondin motifs (*ADAMTS*), was widely reported to be associated with adipogenesis, fat content, and obesity [69–72]. qC20:1ω9, located on LG6, could explain 10.2% of the PVE for C20:1ω9 (Table 3, Additional file 9). One candidate gene identified in this QTL was growth hormone secretagogue receptor (*GHSR*). *GHSR* polymorphisms are associated with carcass traits in sheep, and individuals with a mutant ‘TC’ genotype exhibit greater levels of abdominal fat [73]. For C17:1, the only QTL qC17:1 was located on LG5 with a PVE of 8.9% (Table 3, Additional file 9). The gene identified in this QTL was phospholipid scramblase (*PLSCR*). PLSCR proteins are responsible for the translocation of phospholipids between the two monolayers of a membrane lipid bilayer, and are critical to the normal regulation of lipid metabolism [74–76].

**Fig. 6:**
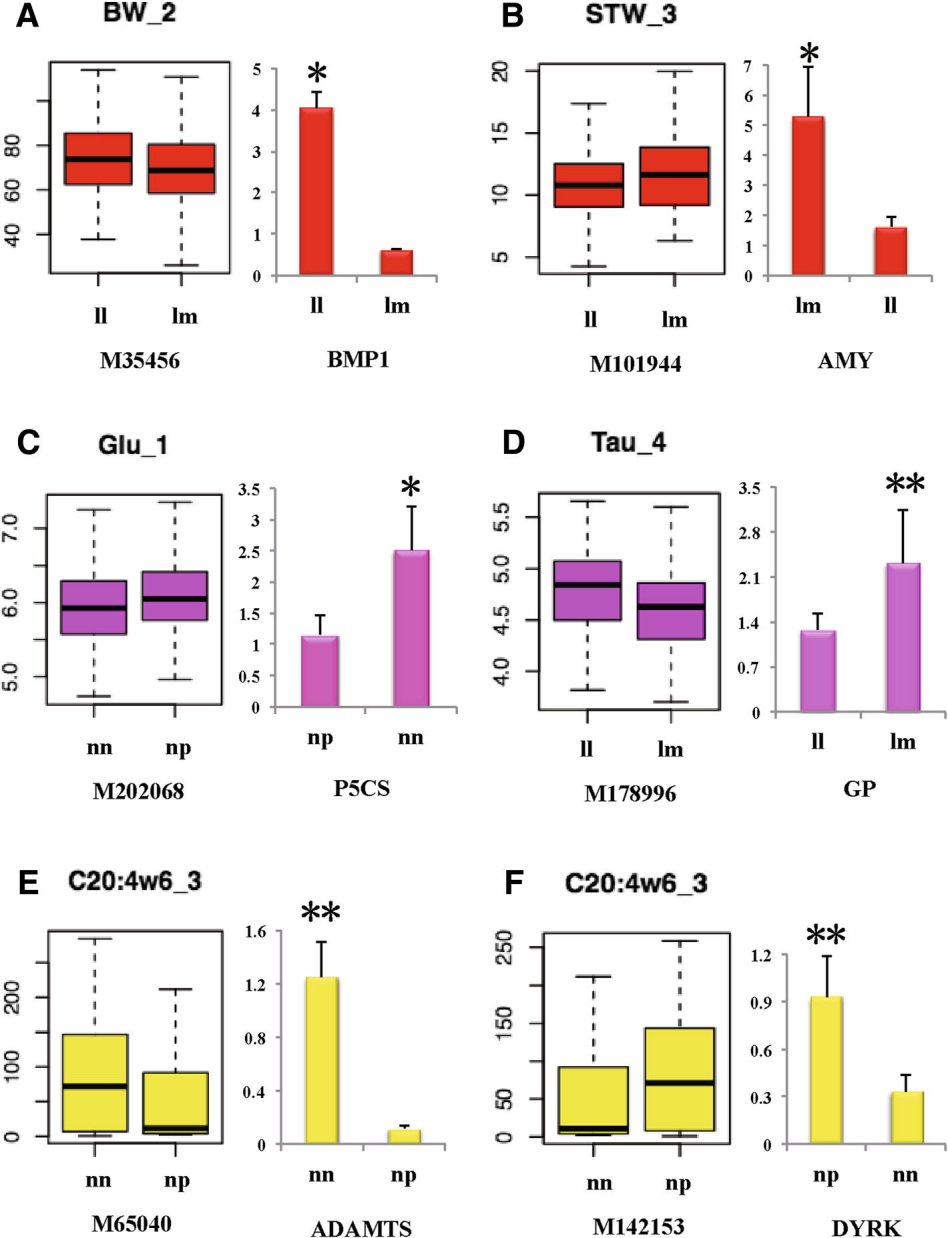
Candidate genes expression analysis under different genotypes and phenotypic values. **a** *BMP1* gene for body weight, **b** *AMY* gene for the weight of the soft tissue, **c** *P5CS* gene for glutamate acid, **d** *GP* gene for taurine, **e** *ADAMTS* gene for C20:4ω6, and **f** *DYRK* gene for C20:4ω6. The left pattern shows the boxplot of phenotypic values under different genotypes of the nearest marker of the candidate gene; while the right pattern shows mRNA expression levels of the candidate gene under different genotypes and phenotypic values

To further study the relationship between the candidate genes and the traits, we analysed the mRNA expression levels among samples with different phenotypic values. Of all 13 identified genes, we designed 9 primers successfully. The results showed that six genes exhibited significant expression difference (*P* < 0.05) between groups with different genotypes and phenotypic values (Fig. 6). Boxplot from Fig. 6 also showed that phenotypic values between different genotypes were partly overlapped with the other, which may reflect the minor effect of single gene to quantitative traits. Further, for the six differently expressed genes, *BMP1* and *AMY* were identified for growth traits, *P5CS* and *GP* were for AA, while *ADAMTS* and *DYRK* were for FA. *P5CS* and *GP* genes showed high mRNA expression levels in ‘low group’, but low mRNA expression levels in ‘high group’; While all the other four genes showed higher mRNA expression levels in ‘high group’.

## Discussion

### High-quality genetic map of *C. gigas*

After applying rigorous filter criteria (missing rate < 10%, segregation distortion *P* < 0.01), 5094 markers were used to construct the genetic map, of which, 5024 (98.62%) were successfully assigned to 10 LGs with the unique marker density of 0.68 cM (Table 1). Furthermore, even marker distribution (Table 1, Fig. 1) and similar patterns of recombination rates between sex-specific maps (Fig. 3) all confirmed the high quality of the consensus map. The Pacific oyster was reported to be of high heterozygosity and polymorphism [77], which may be the main reason for high missing rate for the enzyme-cut markers. It’s intriguing to see that LG6 and LG10 contain almost exclusively paternal and maternal markers, respectively. The Pacific oyster showing widespread segregation distortion in inbred F2 or F3 families, carries a large load of highly deleterious, primarily recessive mutation [78–80]. To some extent, this inbreeding depression was lineage- and environment-specific [81]. Based on which, we speculate that female (sampled from Changli) LG6 and male (sampled from Qingdao) LG10 may bear some structural variation towards the breeding environment, which will cause lethal zygotes and further result in paternal-marker dependent LG6 and maternal-marker dependent LG10. The length of the consensus map was 1982.07 cM, which was longer than previously reported maps with the length ranging from 558.2 to 1084.3 cM [22, 23, 29, 31, 35, 40]. Different mapping methods in JoinMap4.1 may be the major reason causing this difference. Multi-point ML algorithm with haldnane function would increase the map length without considering double recombination events [43]. However, ML algorithm runs more quickly than regression algorithm when there’re more than 400 markers on one LG (a few hours versus more than 1 month) and ML yields better fitting maps than regression method in most cases [35]. Longer map length was also observed in other aquatic species, for which the genetic map was constructed with thousands of markers mainly from NGS [82, 83]. In summary, we have constructed a high-quality genetic map for *C. gigas* with the highest number of markers, the greatest marker density, and the largest genome coverage so far. This highly qualified genetic map will provide a basis for genome assembly and fine mapping of vital economic traits.

### Genome assembly

Recently, high-density genetic maps have been an important tool for genome scaffold assembly of aquatic species. For example, 246 genome scaffold, which represented 95.78% of the assembled genomic sequences of *Paralichthys olivaceus*, were accurately anchored onto 24 LGs by a GBS-based high-resolution genetic map [83]. Similarly, 2839 of 2963 (95.8%) genome scaffold of *Cyprinus carpio* were mapped to unique LGs by a micro-array based high-density genetic map [82]. In this study, all 5024 markers were mapped to 1524 genome scaffold, covering 68.81% of the genome. Further analysis showed that only 13.53% of the scaffold with more than one marker distributed on different LGs of the genetic map (Table 2). Our results not only showed a good collinearity between the consensus map and the oyster genome but also indicated a better genome assembly of *C. gigas*. Accordingly, we successfully assembled a chromosome-level genome framework covering about 70% of the genome sequence of *C. gigas*, providing a key basis for further comparative genomics and fine QTL mapping analysis.

### QTL mapping and identification of candidate genes for growth and nutritional traits

In this study, we utilised a ddGBS-based high-quality linkage map to identify genes controlling both growth and nutritional traits of *C. gigas*. We not only identified known growth-related genes like *AMY* and *BMP1*, but also identified new genes participating in AA and FA metabolism and transportation, like *P5CS*, *rBAT*, *PLSCR*, and *DYRK*. Candidate genes identified for growth were different from the results before [36]. Variation in growth may be influenced by many loci with small effects [31]. Compared with before, different mapping family and enzyme-cut experimental design in this study may produce diverse segregation loci and result in disparate candidate genes. Polymorphism of glycogen phosphorylase was reported to be associated with the glycogen content in *C. gigas* [18], while it was identified in the QTL of Tau in this study. Considering the significant negative correlation between Tau and glycogen (Additional file 5 D), this gene may play a key role in the metabolism regulation of both Tau and glycogen. Genes identified in FA QTLs were reported either to regulate lipid metabolism or to be associated with body weight (Table 4), which was reasonable considering the correlation between fat and body weight. Further, we conducted gene expression analysis for the identified genes. Six genes showed gene expression difference between groups with different genotypes and phenotypic values, which indicated that the gene may influence the trait by transcriptional regulation. Meanwhile, genes identified for AA traits exhibited different expression patterns compared to growth and FA traits, which reflected diverse regulatory mechanism underlying different candidate genes and traits. These six genes will be key targets for molecular breeding of important economic traits. One underlying concern for this study is the overestimation of QTL mapping resulted from low sampling size (Beavis Effect [84]). In this study, we not only conducted strict data filter for each step towards QTL mapping but also verified gene function on transcriptional levels, both of which will convince our findings strongly. Further studies based on the construction of bigger populations or families with more divergent phenotypic difference will also improve the power of QTL mapping.

## Conclusion

In this study, we used ddGBS method to generate a high-density genetic map with the most markers for *C. gigas*. Based on the high collinearity between the linkage map and the oyster genome, we successfully assembled nearly 70% of the oyster genome sequence onto chromosomes for the first time. Besides, we provided valuable markers and candidate genes for growth, and firstly for AA and FA traits of *C. gigas* by QTL mapping. Six genes that can influence the traits by transcriptional regulation may be causative genes for further studies concerning qualified traits improvement. In a whole, our findings will promote genetic dissection and molecular breeding of important economic traits in *C. gigas*.

## Additional files

Additional file 1: Adaptors and primers used in this study. (XLS 34 kb)

Additional file 2: Histogram of growth and nutritional traits. Individual panels show (A) growth and glycogen, (B) amino acid, and (C) fatty acid. Full names for the abbreviation are as follows: shell height (SH), shell length (SL), shell width (SW), body weight (BW), weight of the soft tissue (STW), total amino acids (All_AA), glutamic acid (Glu), taurine (Tau), aspartic acid (Asp), lysine (Lys), alanine (Ala), leucine (Leu), glycine (Gly), proline (Pro), arginine (Arg), valine (Val), serine (Ser), isoleucine (Ile), threonine (Thr), phenylalanine (Phe), tyrosine (Tyr), histidine (His), cysteine (Cys), methionine (Met), total fatty acids (All_FA), C20:5ω3 (EPA), and C22:6ω3 (DHA). (TIF 552 kb)

Additional file 3: Phenotype values and variance. Full names for the abbreviation are shown in Additional file 2. (XLS 112 kb)

Additional file 4: Amino acid and fatty acid composition. (A) Amino acid composition, (B) Fatty acid composition. Full names for the abbreviation are shown in Additional file 2. (TIF 625 kb)

Additional file 5: Correlation analysis of growth and nutritional traits. (A) Correlation analysis of (A) growth and glycogen, (B) amino acid, (C) fatty acid, and (D) representative traits. Digits above the diagonal show correlation coefficient, while patterns below the diagonal show scatter plots of correlation. Significance correlation (*P* < 0.05) and (*P* < 0.01) was indicated by * and **, respectively. Full names for the abbreviation are shown in Additional file 2. (TIF 2998 kb)

Additional file 6: Missing rate statistics for all polymorphic markers of the mapping family. (XLS 25 kb)

Additional file 7: Detailed information of 5024 haplotype markers used for genetic map construction. (XLS 12914 kb)

Additional file 8: Summary of the sex-specific maps. (XLS 35 kb)

Additional file 9: QTL mapping for growth and nutritional traits. Full names for the abbreviation are shown in Additional file 2. (TIF 2942 kb)

## Abbreviations

AA: Amino acid
Ala: Alanine
All_AA: Total amino acids
All_FA: Total fatty acids
Arg: Arginine
Asp: Aspartic acid
BW: Body weight
Cys: Cysteine
DHA: C22:6ω3
EPA: C20:5ω3
FA: Fatty acid
Glu: Glutamic acid
Gly: Glycine
His: Histidine
Ile: Isoleucine
Leu: Leucine
Lys: Lysine
Met: Methionine
Phe: Phenylalanine
Pro: Proline
QTL: Quantitative trait loci
Ser: Serine
SH: Shell height
SL: Shell length
STW: Weight of the soft tissue
SW: Shell width
Tau: Taurine
Thr: Threonine
Tyr: Tyrosine
Val: Valine
