# Supplementary figures and images for: Construction of a high-density genetic map and fine QTL mapping for growth and nutritional traits of Crassostrea gigas
Source: BMC Genomics. 2018 Aug 22;19:626. doi: 10.1186/s12864-018-4996-z (PMC6106840; doi:10.1186/s12864-018-4996-z)

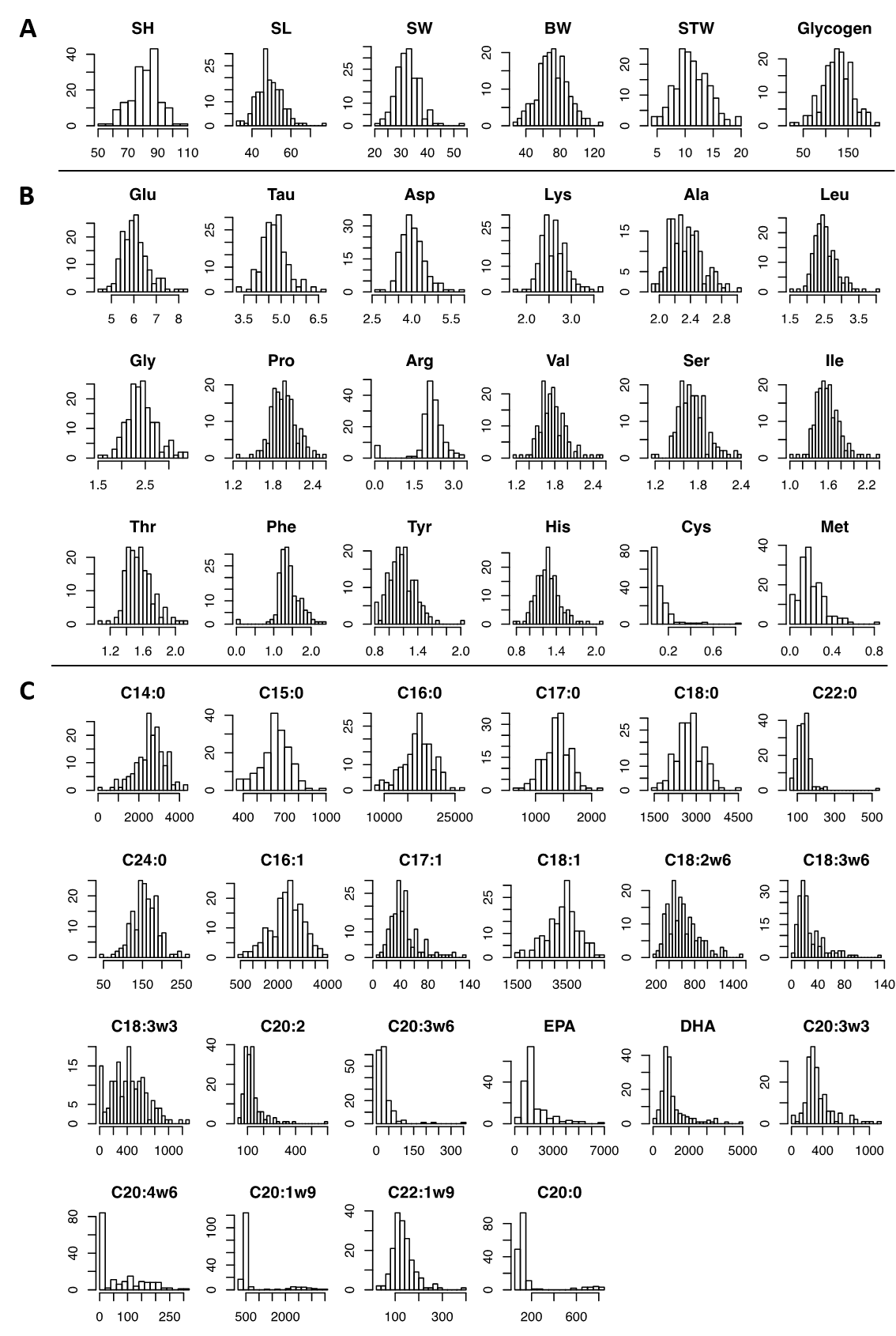

Supplement: Supplementary file 2 — Histogram of growth and nutritional traits. Individual panels show (A) growth and glycogen, (B) amino acid, and (C) fatty acid. Full names for the abbreviation are as follows: shell height (SH), shell length (SL), shell width (SW), body weight (BW), weight of the soft tissue (STW), total amino acids (All_AA), glutamic acid (Glu), taurine (Tau), aspartic acid (Asp), lysine (Lys), alanine (Ala), leucine (Leu), glycine (Gly), proline (Pro), arginine (Arg), valine (Val), serine (Ser), isoleucine (Ile), threonine (Thr), phenylalanine (Phe), tyrosine (Tyr), histidine (His), cysteine (Cys), methionine (Met), total fatty acids (All_FA), C20:5ω3 (EPA), and C22:6ω3 (DHA). (TIF 552 kb) [file 12864_2018_4996_MOESM2_ESM.tif]

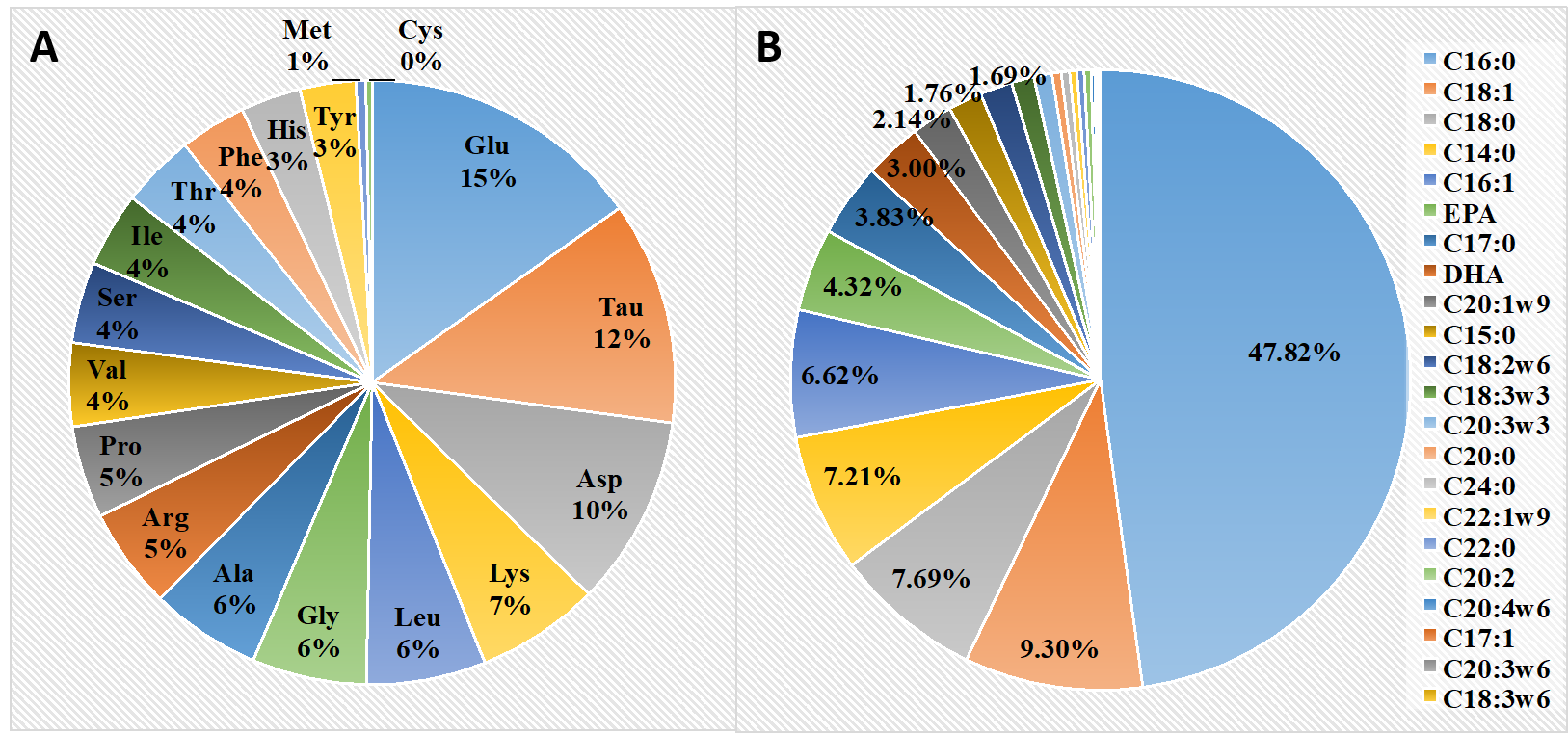

Supplement: Supplementary file 4 — Amino acid and fatty acid composition. (A) Amino acid composition, (B) Fatty acid composition. Full names for the abbreviation are shown in Additional file 2. (TIF 625 kb) [file 12864_2018_4996_MOESM4_ESM.tif]

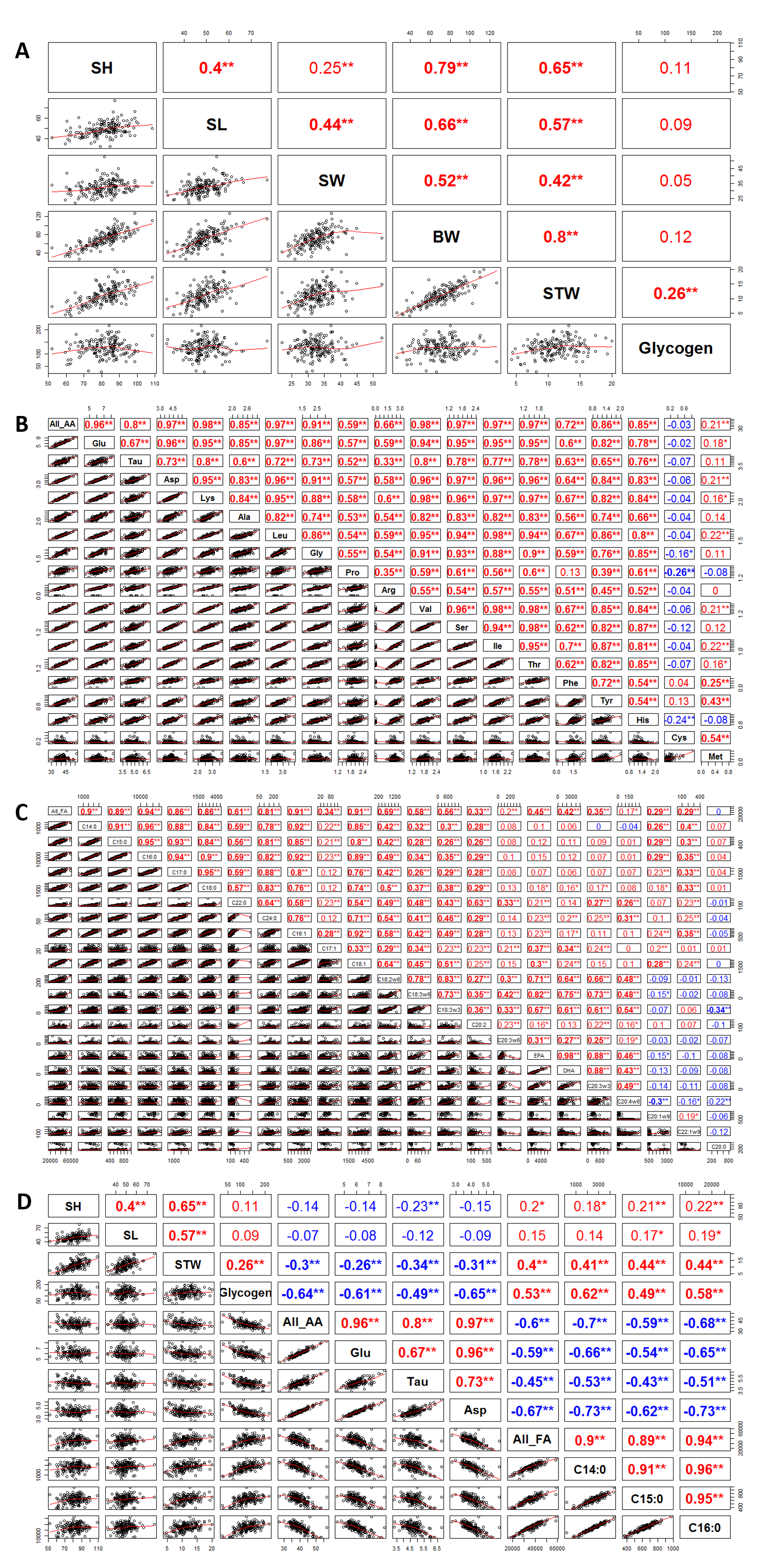

Supplement: Supplementary file 5 — Correlation analysis of growth and nutritional traits. (A) Correlation analysis of (A) growth and glycogen, (B) amino acid, (C) fatty acid, and (D) representative traits. Digits above the diagonal show correlation coefficient, while patterns below the diagonal show scatter plots of correlation. Significance correlation (P < 0.05) and (P < 0.01) was indicated by * and **, respectively. Full names for the abbreviation are shown in Additional file 2. (TIF 2998 kb) [file 12864_2018_4996_MOESM5_ESM.tif]
